# Dietary creatine and kidney function in adult population: NHANES 2017–2018

**DOI:** 10.1002/fsn3.2200

**Published:** 2021-02-20

**Authors:** Sergej M. Ostojic

**Affiliations:** ^1^ FSPE Applied Bioenergetics Lab University of Novi Sad Novi Sad Serbia

**Keywords:** creatine, creatinine, diet, kidney function, populational study

## Abstract

Consuming more creatine may be associated with an increased risk of renal dysfunction, yet this link remains poorly addressed at the population level. Using 2017–2018 NHANES data, the current study found that the odds ratio for having failing kidneys in 2,955 U.S adults consuming ≥2.0 g/day of dietary creatine compared to low‐intake counterparts (<1.0 g/day) was 0.74 (95% CI from 0.39 to 1.38), indicating no significant association between dietary creatine intake and kidney dysfunction.

## Graphical abstract

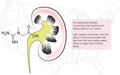

## 1 INTRODUCTION

Creatine is a conditionally essential nutrient that is heavily involved in human energy metabolism. It serves as a spatial and temporal energy buffer for many organs with high energy needs, including the brain, skeletal muscle, kidney, and liver (Wallimann et al. 2011). A daily turnover of creatine is approximately 2.0 grams, and about half of this daily need for creatine (1.0 g/day) is obtained from the diet, while the rest is de novo synthesized inside the body. Creatine is generally considered a safe nutritional compound (Balestrino & Adriano, 2019), yet several case reports suggest that high levels of creatine in a diet may compromise kidney function (Taner et al. 2011; Thorsteinsdottir et al. 2006). In contrast, a plethora of randomized controlled studies reported no damaging effects of dietary creatine on renal function (for a detailed review see Souza et al., 2019). Still, a possible association between the risk of renal dysfunction and dietary creatine remains poorly addressed at the population level. In this cross‐sectional study, the risk of kidney failure in U.S. adults consuming different amounts of dietary creatine was evaluated, using data from the 2017–2018 National Health and Nutrition Examination Survey (NHANES) round.

## 2 METHODS

The NHANES population includes the noninstitutionalized civilian residents of the United States. The cohort of NHANES 2017–2018 comprised a total of 9,245 male and female respondents aged 0 – 80 years. For this analysis, we sorted out data for adult respondents (aged 20 years and over) who provided information on dietary intake and kidney function. Dietary intake information was obtained through dietary 24‐hr recall interviews. Individual data files containing detailed information about each food/beverage item (including the description, amount of, and nutrient content) were used to calculate creatine intake from meat‐based protein foods as previously described (Bakian et al. 2020). Afterward, the respondents were categorized into three groups of daily creatine intake: low‐intake group (creatine intake < 1.0 g/day), medium‐intake group (1.00 – 1.99 g/day), and high‐intake group (≥ 2.0 g/day), with the medium‐intake group excluded from the inter‐group comparison. This margin was chosen due to the fact that most adults consume 1.0 gram of dietary creatine per day, which is considered a recommended amount (Brosnan et al., 2011). The information about kidney function was extracted from NHANES 2017–2018 Questionnaire Data on kidney conditions and urology codebook. Kidney dysfunction was determined for participants who positively responded to the question has he/she ever been told by a doctor or other health professional that had weak or failing kidneys (excluding kidney stones, bladder infections, or incontinence). Data collected from the NHANES 2017–2018 laboratory domain were acquired to identify relevant variables for kidney function, including blood urea nitrogen and serum creatinine, and urinary flow rate, creatinine, and albumin‐to‐creatine ratio. Serum and urine samples were processed, stored, and analyzed at the University of Minnesota Advanced Research Diagnostics Laboratory. Serum variables and urinary creatinine were measured on the Roche Cobas 6,000 analyzer (Indianapolis, IN), with detailed instructions on specimen collection and processing available in the NHANES laboratory procedures manual (NHANES, 2017). The NHANES quality assurance and quality control protocols were in accordance with the 1988 Clinical Laboratory Improvement Act mandates. The approval to conduct NHANES 2017–2018 was granted by the U.S. National Center for Health Statistics Research Ethics Review Board (#2018‐01 and #2011‐17). NHANES complex sampling design was employed for data management. Data series were first analyzed by the Kolmogorov‐Smirnov test for normality of distribution. Independent Mann–Whitney U test and chi‐square tests were employed to compare mean values and proportions across two categories, respectively. The odds ratio was calculated to quantify the strength of the association between dietary creatine intake and kidney failure in two subpopulations. Data were analyzed using IBM SPSS Statistics for Mac (Version 24.0), with the significance level set at *p* <.05.

## 3 RESULTS AND DISCUSSION

A total NHANES 2017–2018 cohort of U.S. adults who provided information on dietary intake of creatine and kidney function was 3,995 (1,930 men and 2,065 women). The mean daily intake of creatine was 0.94 ± 0.77 (95% confidence interval [CI] from 0.92 to 0.96), and 170 participants (4.26%) reported kidney failure across the whole sample. After we excluded the participants who reported medium intake of creatine, the final sample contained 2,955 respondents with either low intake (*n* = 2,606) or high intake (*n* = 349) of dietary creatine. The profiles of these two subpopulations are presented in Table 1. Except for the significantly higher dietary creatine intake found in the high‐intake group (2.78 ± 0.86 g/day versus. 0.52 ± 0.26 g/day; *p* <.001), no differences were found among the two groups for other variables, including kidney failure prevalence and mean values for selected biomarkers of kidney function (*p* >.05). In addition, the odds ratio for having failing kidneys in U.S adults consuming ≥2 g/day of dietary creatine compared to low‐intake peers (<1 g/day) was 0.74 (95% CI from 0.39 to 1.38), indicating no significant association between dietary creatine intake and kidney dysfunction.

**TABLE 1:** Demographics and biochemical profiles of U.S. adults with low and high intake of creatine

Variable	Low intake		High intake		*p*
	Mean ± *SD*	*n*	Mean ± *SD*	*n*	
Age (years)	51.3 ± 17.9	2,606	51.9 ± 17.5	349	.586
Gender, *F* (%)	47.9	2,606	46.4	349	.596
Body mass index (kg/m^2^)	26.6 ± 8.3	2,396	27.3 ± 8.6	324	.186
Kidney failure (%)	4.2	2,606	3.2	349	.344
Blood urea nitrogen (mg/dL)	14.7 ± 6.1	2,413	14.7 ± 6.3	322	.817
Serum creatinine (mg/dL)	0.88 ± 0.41	2,414	0.87 ± 0.29	322	.832
Urinary creatinine (mg/dL)	126.5 ± 86.1	2,507	122.7 ± 83.1	338	.462
Urinary albumin‐to‐creatinine ratio (mg/g)	49.2 ± 364.2	2,507	66.9 ± 415.5	338	.276
Urinary flow rate (mL/min)	0.91 ± 1.28	1,921	1.00 ± 3.18	257	.696

This population‐level study revealed no relationship between consuming more creatine and kidney failure in U.S. adults. It appears that high‐creatine consumers, who eat about 5 times more creatine per day than their low‐creatine peers, show no higher risk of kidney failure. Besides, the odds ratio of 0.74 tends to favor a decreased occurrence of an event in the high‐intake group, suggesting a protective outlook of consuming 2.0 grams or more creatine per day.

A case for dietary creatine‐induced kidney damage has largely been built as a consequence of several case reports, including the patient with genetic mitochondrial disease (Barisic et al. 2002), an athlete who consumed 52 (!) food supplements, including creatine (Thorsteinsdottir et al. 2006), and hypertensive women supplemented with questionable creatine product (Fages et al. 2019). Those and similar case studies put forward the possibility for adverse effects of dietary creatine on renal function, justifying medical attention especially in patients with pre‐existing nephropathies. However, far more studies demonstrated no effects of dietary creatine on kidney function in both athletic and clinical environment, with a recent meta‐analysis with 497 subjects and 15 studies (11 longitudinal trials and 4 case studies) concluded that creatine consumption does not alter serum creatinine and urea levels (Souza et al., 2019). Post et al. (2019) even suggested that additional creatine from food might be necessary for patients with chronic kidney disease, to safely support the lower endogenous production of creatine and the unopposed losses of creatine in this condition. These opposing assertions from previous studies are accompanied by a vast paucity of populational data exploring the connection between dietary creatine and kidney damage. To the author's best knowledge, the present study is the first survey that examined this link in a case‐control design at the level of population. The current report found no association between dietary creatine and kidney failure, accompanied by matching values for biomarkers of kidney function in two contrasting categories of creatine intake. This perhaps indicates that taking more creatine (≥2 g/day) induces no weak kidneys, which mostly corroborates findings from previous interventional trials. Still, the question remains whether higher doses of dietary creatine (*e.g*., above 5 g/day) increase the risk of kidney damage at the populational level. In the NHANES 2017–2018 cohort, we found only 5 adults (all men) that were calculated to have a dietary intake of creatine above 5 grams per day, which is too undersized to conduct any risk assessment. Nevertheless, no single individual from this super high‐intake group reported kidney failure and has biochemical markers above the normal values.

The present study limitations include the following: (a) the premise that kidney failure information acquired from kidney conditions questionnaire provides a legitimate estimate of renal function; (b) the lack of additional biomarkers (*e.g*., plasma cystatin C, symmetric dimethylarginine, iohexol clearance) that could provide more detailed information about kidney (dys)function after an exposure (Ostojic, 2020); (c) the method of collecting dietary intake data that depends on self‐reported information, instead of using independent techniques, such as the doubly labeled water or the 24‐hr urine nitrogen output; (d) the omission to count for nonmeat foods and beverages as dietary sources of creatine, although those foods provide very little creatine (Hülsemann et al., 1987); and (e) setting a somewhat arbitrary threshold of low to high intake of creatine. Future populational studies should continue to monitor kidney function in the general public exposed to creatine using advanced approaches, across various age groups and dietary creatine ranges, and validate our findings that underscore no connection between kidney failure and food creatine.

## CONFLICT OF INTEREST

SMO serves as a member of the Scientific Advisory Board on creatine in health and medicine (AlzChem LLC). SMO owns patent "Sports Supplements Based on Liquid Creatine" at European Patent Office (WO2019150323‐A1), and active patent application "Synergistic Creatine" at UK Intellectual Property Office (GB2012773.4). SMO has served as a speaker at Abbott Nutrition, a consultant of Allied Beverages Adriatic and IMLEK, and an advisory board member for the University of Novi Sad School of Medicine, and has received research funding related to creatine from the Serbian Ministry of Education, Science, and Technological Development, Provincial Secretariat for Higher Education and Scientific Research, AlzChem GmbH, KW Pfannenschmidt GmbH, and ThermoLife International LLC. SMO is an employee of the University of Novi Sad. SMO does not own stocks and shares in any organization.

## STUDIES INVOLVING HUMAN SUBJECTS

The study conforms to the Declaration of Helsinki for human subjects.
